# The links between sleep duration, obesity and type 2 diabetes mellitus

**DOI:** 10.1530/JOE-21-0155

**Published:** 2021-11-12

**Authors:** Christina Antza, Georgios Kostopoulos, Samiul Mostafa, Krishnarajah Nirantharakumar, Abd Tahrani

**Affiliations:** 1 Institute of Metabolism and Systems Research, University of Birmingham, Birmingham, UK; 2 Department of Endocrinology, 424 General Military Hospital, Thessaloniki, Greece; 3 Department of Diabetes and Endocrinology, University Hospitals Birmingham NHS Foundation Trust, Birmingham, UK; 4 Institute of Applied Health Research, University of Birmingham, Birmingham, UK; 5 Centre of Endocrinology Diabetes and Metabolism, Birmingham Health Partners, Birmingham, UK

**Keywords:** obesity, diabetes mellitus, sleep duration, long sleep, short sleep, sleep deprivation, sleep manipulation, type 2 diabetes, sleep

## Abstract

Global rates of obesity and type 2 diabetes mellitus (T2DM) are increasing globally concomitant with a rising prevalence of sleep deprivation and sleep disorders. Understanding the links between sleep, obesity and T2DM might offer an opportunity to develop better prevention and treatment strategies for these epidemics. Experimental studies have shown that sleep restriction is associated with changes in energy homeostasis, insulin resistance and β-cell function. Epidemiological cohort studies established short sleep duration as a risk factor for developing obesity and T2DM. In addition, small studies suggested that short sleep duration was associated with less weight loss following lifestyle interventions or bariatric surgery. In this article, we review the epidemiological evidence linking sleep duration to obesity and T2DM and plausible mechanisms. In addition, we review the impact of changes in sleep duration on obesity and T2DM.

## Introduction

Obesity prevalence increased globally between 1975 and 2016, from 0.7 to 5.6% in girls, from 0.9 to 7.8% in boys and from 4.7 to 13.1% in adults with big variations amongst different world regions (Abarca-Gómez *et al.* 2017) (https://apps.who.int/gho/data/view.main.REGION2480A?lang=en Accessed (September) (2021)). Considering the health and economic consequences associated with obesity (type 2 diabetes mellitus (T2DM), cardiovascular disease (CVD), cancer, mortality), there is huge interest in strategies to reduce obesity prevalence (Guh *et al.* 2009, Lu *et al.* 2014, Blackman *et al.* 2016, Zheng *et al.* 2016, Asad *et al.* 2018, Mistry *et al.* 2018, Singh *et al.* 2020) (https://openknowledge.worldbank.org/bitstream/handle/10986/32383/211491ov.pdf?sequence=4&is%20Allowed=y Accessed (September) (2021)) (https://www.weforum.org/agenda/2019/10/obesity-healthcare-expenditure-burden/ Accessed (September) (2021)).

Similar to the increase in obesity prevalence, T2DM prevalence has also increased dramatically over the last three decades, and it is predicted to reach 11% globally by 2045 (Saeedi *et al.* 2019) (https://www.who.int/news-room/fact-sheets/detail/diabetes Accessed (September) (2021)). Obesity is associated with an increased risk of T2DM (Kahn *et al.* 2006). Similar to obesity, T2DM has a major negative health (CVD, diabetes-related microvascular complications, mortality) and economic impact (Stamler *et al.* 1993, Brownrigg *et al.* 2016) (https://www.nicswell.co.uk/health-news/diabetes-cases-and-costs-predicted-to-rise Accessed (February) (2021)). Hence, T2DM prevention strategies are of paramount importance, and addressing obesity is key to reduce the burden of this disease (Jones *et al.* 2006, Milstein *et al.* 2007).

Despite multiple approaches including policy (sugar tax for example), lifestyle interventions and pharmacological and non-pharmacological treatments, to contain this syndemic, the prevalence of obesity and T2DM continued to increase globally and no country has managed to reduce the prevalence of either disease. Hence, there is a need for a better understanding of the complex disease pathogenesis and improved understanding of the modifiable risk factors to reduce the burden of obesity and T2DM.

Further to the increases in the obesity and T2DM prevalence, sleep insufficiency has become part of the modern lifestyle, across all age groups (Gangwisch *et al.* 2005, Basch *et al.* 2014, Owens *et al.* 2014, Gordon *et al.* 2019, Toyoura *et al.* 2020). The average sleep duration dropped from 8–9 h/night in 1960 to 7 h/night in 1995 (Van Cauter & Knutson 2008). The prevalence of short sleep duration was reported as 45% in the United States in 2014, with a third of US adults not getting enough sleep (<7 h/night), which led the Centre for Disease Control in the United S to labelling sleep insufficiency as a public health epidemic (https://www.aaas.org/news/sleep-deprivation-described-serious-public-health-problem. Accessed (September) (2021)). This is further emphasised by the associations between short sleep and adverse health outcomes, including CVD and mortality among others (https://www.cdc.gov/sleep/data_statistics.html Accessed (September) (2021)). Hence, improving sleep duration might be an important strategy to reduce the prevalence and economic impacts of obesity and T2DM. However, despite its potential importance, sleep disorders are largely underdiagnosed.

The relationship between sleep duration, obesity and T2DM is likely to be bi-directional. Obesity and T2DM can disrupt sleep due to the high prevalence of obstructive sleep anoea or the occurrence of hypoglycaemia or nocturnal symptoms such nocturia or painful neuropathy (Surani *et al.* 2015, Lavrentaki *et al.* 2019). In addition, changes in body weight could lead to changes in sleep duration. Clinically significant weight loss was found to be associated with decreased daytime sleepiness and time to fall asleep, and increased sleep duration in people with short sleep duration (≤7 h) (Verhoef *et al.* 2013). In this article, we critically review only the evidence linking sleep duration to obesity and T2DM, with a particular focus on the epidemiology and interventional studies to examine whether manipulating sleep duration can be utilised as a strategy to prevent or treat obesity and T2DM. We will also review recent evidence regarding the mechanisms linking sleep duration to obesity and T2DM.

## Methods

We performed a scoping literature search using Medline, the Cochrane Library, CINAHL and PsycINFO from inception to 31 August 2021, to identify studies that examined the relationship between sleep duration and obesity as well as sleep duration and T2DM (PICO question is presented Supplementary content 1, see section on supplementary materials given at the end of this article). We used appropriate search terms that had been identified from initial scoping searches, target references, and browsing of database thesauruses (sleep duration and diabetes (Supplementary content 2). A basic search strategy was developed for PubMed and modified accordingly for other research engines. This review will mainly focus on cohort and interventional studies. We utilized meta-analyses and systematic reviews where available and summarised the evidence that was not included in the latest systematic reviews.

## Assessing sleep duration

Self-reported sleep duration is widely used in research and clinical practice due to its ease of use and the ability to apply it to large population. However, the increasing availability of modern technology aiding better assessments of sleep highlighted the shortcomings of self-reported sleep duration. Self-report does not reflect the objectively measured sleep. A study of more than 5000 participants, aged more than 65 years old, showed a poor agreement between self-reported and actigraphic sleep duration (Kappa ≤ 0.24). Nearly half of the patients who reported normal sleep had short sleep on actigraphy and more than a quarter of participants that reported short sleep had normal sleep on actigraphy (Miner *et al.* 2021). These discrepancies between subjective and objective measures of sleep duration were observed in multiple ethnicities including Whites, Blacks, Hispanics and Chinese (Jackson *et al.* 2018). In another study of 2086 participants in the United States, self-reported sleep duration was significantly greater than actigraphy-measured sleep duration (7.85 ± 1.12 h vs 6.74 ± 1.02 h, respectively) (Cespedes *et al.* 2016*b*). Correlations between self-reported and actigraphy-assessed time spent asleep were lower with male sex, younger age, sleep efficiency <85% and night-to-night variability in sleep duration ≥1.5 h.

Hence, the limitations of self-reported sleep duration need to be considered when interpreting the studies listed in this review.

## Epidemiological studies

### Sleep duration and obesity

#### Sleep duration as a risk factor for obesity

Cross-sectional studies showed conflicting results regarding the relationship between sleep duration and obesity ranging from linear or U-shaped associations to no relationship (Yan *et al.* 2017, Rosique-Esteban *et al.* 2018, Park *et al.* 2020). This difference can be in part due to the impact of age on the relationship between sleep duration and obesity and differences in the methods of assessing sleep duration or obesity (objective vs subjective) (Yan *et al.* 2017, Rosique-Esteban *et al.* 2018, Park *et al.* 2020). The association between short sleep and obesity is stronger in children compared to adults and in younger adults compared to older adults. Variation regarding this association could also be attributed to different anthropometric measurements applied in obesity definitions, such as BMI, waist circumference (WC) and abdominal adiposity.

Unlike cross-sectional studies, the results of cohort studies have been more consistent. Multiple meta-analyses of cohort studies in the last 10 years examined also the relationship between sleep duration and the risk of developing obesity in adults and children (Table 1) (Wu *et al.* 2014, 2017, Capers *et al.* 2015, Fatima *et al.* 2015, Ruan *et al.* 2015, Sperry *et al.* 2015, Itani *et al.* 2017, Li *et al.* 2017, Jike *et al.* 2018, Deng *et al.* 2021, Guo *et al.* 2021, Miller *et al.* 2021).

**Table 1:** Ten years examining the relationship between sleep duration and obesity.

Study	Number of studies	Number of participants	Age	Sleep recordings	Sleep duration	Comparator	Range of follow-up (years)	Outcome (assessment method)	95% CI	I^2^	Adjustment for covariates
Adults cohorts											
Wu *et al.* 2014	14	197,906	≥18	Self-reported	Short sleep (≤6 h)	Normal (7–8 h)	1–12	Obesity (BMI):OR = 1.45	1.25, 1.67	66.2	1, 2, 3, 4, 5, 6, 7, 8, 9, 10, 11, 12, 13, 14, 15, 16, 17, 18, 19, 28
Wu *et al.* 2014	10	164,016	≥18	Self-reported	Long sleep (>7 h)	Normal (7 h)	1–12	Obesity (BMI):OR = 1.06	0.98, 1.15	0	
Itani *et al.* 2017	16	322,842	≥18	Self-reported, objectively	Short sleep (<7 h)	Normal (7–8 h)	1–30	Obesity (BMI/WC): RR = 1.38	1.25, 1.53	60	1, 2, 3, 4, 5, 6, 7, 8, 9, 10, 11, 12, 13, 14, 15, 16, 17, 18, 19, 28
Jike *et al.* 2018	13	318,437	≥18	Self-reported, objectively	Long sleep (>7 h)	Normal (6–8)	1– 34	Obesity (N/A):RR = 1.08	1.02, 1.15	0	
Bacaro *et al.* 2020	12	154,936	≥18	Self-reported, objectively	Short sleep (≤6.5 h)	Normal (6–8)	1–12	Obesity (BMI):OR = 1.41	1.18, 1.69	80	1, 2, 4, 5, 6, 7, 8, 14
Bacaro *et al.* 2020	8	152,192	≥18	Self-reported	Long sleep (>8 h)	Normal (7–8)	1–12	Obesity (BMI):OR = 0.99	0.89, 1.11	0	
Zhou *et al.* 2019	12	198,579	≥18	Self-reported, objectively	Short sleep (<7 h)	Normal (7–8)	1–14	Obesity (BMI): RR = 1.48	1.29, 1.72	66.3	1, 2, 3, 4, 5, 6, 7, 8, 9, 10, 11, 12, 13, 14, 15, 16, 17, 18, 19, 28
Zhou *et al.* 2019	9	196,041	≥18	Self-reported, objectively	Long sleep (>8 h)	Normal (7–8)	1–14	Obesity (BMI):RR = 1.04	0.96,1.13	0	
Children cohorts											
Li *et al.* 2017	12	44,200	≤18	Self/parents-reported, objectively	Short sleep*	Normal*	2–15	Obesity (BMI):RR = 1.45	1.14, 1.85	94.6	1, 2, 34, 7, 8, 11, 13, 14, 15, 19, 20, 21, 22, 23, 24, 25, 28, 31
Fatima *et al.* 2015	11	24,821	≤18	Self-reported, objectively	Short sleep *	Normal*	1–27	Overweight/obesity (BMI): OR = 2.15	1.64, 2.81	67	N/A
Ruan *et al.* 2015	10	14,879	≤16	Self/parents-reported	Shortest sleep duration (N/A)	Longest sleep duration (N/A)	0.5–10	Overweight/obesity (BMI):OR = 1.76	1.39, 2.23	70	1, 2, 3, 4, 7, 8, 11, 13, 14, 15, 20, 21, 22, 23, 24, 26, 27, 28, 29, 31
Wu *et al.* 2017	15	35,540	≤18	Self/parents-reported	Short sleep *	Normal*	1–27	Obesity (BMI):OR = 1.71	1.36, 2.14	91.3	1, 2, 3, 4, 8, 11, 13, 14, 20, 22, 23, 24, 28, 29, 30, 31
Guo *et al.* 2021	3	17,038	≤20	Self/parents-reported	Shorter sleep duration	Longer Sleep duration	2–6	Obesity (BMI):RR = 1.40	1.01, 1.95	59	1, 2, 8, 20, 21, 23, 24, 32, 34
Guo *et al.* 2021	5	32,607	≤20	Self/parents-reported	Shorter sleep duration	Longer Sleep duration	1.5–6	Overweight/obesity (BMI):RR = 1.47	1.26, 1.71	1	1, 2, 5, 6, 8, 9, 13, 20, 21, 23, 24, 32, 34
Miller *et al.* 2021	13	42,878	<8	Self/parents-reported, objectively	Shorter* sleep duration	Longer* sleep duration	1–10.25	Overweight/obesity (BMI):RR = 1.54	1.33, 1.77	68	1, 2, 3, 8, 9, 11, 13, 20, 21, 23, 24, 26, 28, 30, 31
Deng *et al.* 2021	29	85,132	<16	Self/parents-reported, objectively	Shorter* sleep duration	Longer* (+normal) sleep duration	1–27	Obesity (BMI):RR = 1.57	1.36, 1.81	91.9	1, 2, 3, 48, 9, 21, 23, 24, 32, 34, 35
Deng *et al.* 2021	9	25,836	2–12	Self/parents-reported, objectively	Longer* Sleep duration	Shorter* (+normal) sleep duration	1–10.25	Obesity (BMI):RR = 0.83	0.75, 0.93	39	

Adjustment for covariates**:** (1) age, (2) sex, (3) race, (4) baseline BMI, (5) smoking, (6) alcohol consumption, (7) caffeine consumption, (8) physical activity, (9) socio-economic status (income, health insurance status, employment status, education, marital status), (10) shift working, (11) eating habits, (12) comorbidities (hypertension, dyslipidaemia, myocardial infraction, cerebral infraction, diabetes), (13) total calorie intake, (14) depression and other mental disorders, (15) sleep-related disorders (insomnia, snoring), (16) use of hypnotic drugs, (17) postmenopausal hormone medications, (18) family history of obesity, (19) study level, (20) birth weight, (21) birth order-parity, (22) environmental (urban residence, country), (23) breastfeeding duration, (24) parental factors (education, income, marital status, smoking or alcohol consumption, maternal pregnancy BMI and age, obesity status), (25) tonsillectomy and/or adenoidectomy, (26) prematurity, (27) season of birth, (28) television watching, (29) weight gain, (30) naptime, (31) late cereal introduction, (32) appetite, (33) accelerometer wear time, (34) school clustering, (35) change in sleep duration.

*Recommended short sleep cut-off values varied according to children’s age.

I^2^, heterogeneity; N/A, not available; WC waist circumference.

All the meta-analyses (whether adults or children) of cohort studies had large sample sizes (from 15,000 to 200,000). The length of follow-up varied from 0.5 to 34 years. Sleep duration was mostly self-reported (or parent-reported in children studies). Two meta-analyses performed sensitivity analyses excluding studies in which sleep duration was objectively measured (Bacaro *et al.* 2020, Zhou *et al.* 2020). To our knowledge, there is not any available meta-analysis to use exclusively normal weight children in the eligibility criteria in order to identify the possibility of obesity in adulthood. However, the outcomes were adjusted to baseline BMI.

Based to cohort-included meta-analytic data, short sleep duration has been consistently associated with increased risk of obesity (whether defined based on BMI or WC). On the other hand, the association between long sleep duration and obesity was not consistent and some meta-analyses showed no associations. Multiple definitions of short sleep duration were used, but all definitions (<7, <6, <5, <4 h/night) were associated with increased risk of obesity in adults (Itani *et al.* 2017). The shorter the sleep duration, the higher the risk of obesity; the pooled relative risk (RR) for obesity was 1.09 (95% CI, 1.05–1.14) per 1-h decrement of sleep duration compared to 7–8 h (Itani *et al.* 2017, Zhou *et al.* 2019). The association between sleep duration and obesity was consistent among multiple subgroups analyses, such as age, gender, ethnicity, follow-up time, quality of study and BMI cut-offs. Notably, sleep duration seemed to have a greater impact on adiposity in female gender. The latter might be attributed to different hormonal profile and socioeconomic status (Zhou *et al.* 2020).

In addition, people with short sleep duration or poor sleep quality lost less fat mass, while having a 600 kcal restricted diet during a longitudinal study over 24 weeks (Chaput & Tremblay 2012). For each additional hour of sleep, fat loss increased by 0.77% or 0.72 kg. Moreover, data from a small pilot study (14 patients who underwent bariatric surgery) highlight the role of sleep in long-term weight loss maintenance at 6 and 9 years post-surgery (Zuraikat *et al.* 2019). Sleep duration was inversely related to BMI at 6‐year follow‐up (R^2^ = 0.41, adjusted R^2^ = 0.36, *P*  = 0.02) but not at 9‐year follow‐up (R^2^ = 0.27, adjusted R^2^ = 0.21, *P*  = 0.055) suggesting that short sleep was associated with greater weight regain after bariatric surgery.

Hence, short sleep not only might increase the risk of developing obesity but might also affect the outcome of weight loss interventions. Whether sleep manipulation when combined with weight loss interventions enhance the outcomes of these interventions need to be examined in randomised controlled trials (RCTs).

#### The relationships between changes in sleep duration and obesity

Although short sleep duration is an independent risk factor for obesity, it is important to examine whether changes in sleep duration could mitigate the burden of obesity or not, but such evidence is limited. In a small study with a follow-up duration of 6 years, based on the cohort of Quebec Family Study, patients with short sleep duration at baseline (≤6 h/night, *n*  = 43) were categorised into two groups: those who increased their sleep duration to 7–8 h/night (mean increase 1.52 ± 0.66 h/day) and those who did not altered their short sleep duration over the follow-up. The control group in this study was 173 people with 7–8 h/night sleep duration. Over the 6 years follow-up, the control group had the smallest increase in BMI (mean (s.e.) 0.8 ± 0.15 kgm^−2^), fat mass (mean (s.e.) 0.75 ± 0.35 kg) and waist circumference (mean (s.e.) 2.4 ± 0.45 cm) compared to the other two groups (approximation with WebPlotDigitizer 4.4 for Mac). Short sleepers who did not increase their sleep duration had greater increase in BMI (mean difference 1.1 ± 0.36 kg m^−2^, *P* < 0.05), fat mass (2.4 ± 0.64 kg, *P* < 0.05) and waist circumference (mean difference 1.4 ± 1.1 cm (approximation with WebPlotDigitizer 4.4 for Mac), *P*  = 0.09) compared to short sleepers who increased their sleep duration, after adjusting for age, sex, baseline BMI, smoking habits, energy intake and physical activity (Chaput *et al.* 2012). The increase in adiposity measures in those with short sleep that increased their sleep duration was similar to the control group (Chaput *et al.* 2012). The sleep duration in this study was self-reported, and the reasons for sleep change were not examined. This is important as the ‘trigger’ to change sleep duration might be related to the changes in BMI (Chaput *et al.* 2012).

However, the Nurses Health Study showed that prolonging self-reported sleep by ≥1 h/ day was associated with weight gain (Cespedes *et al.* 2016*a*). The Nurses Health Study followed up 59,013 women aged 55–83 years between 1986 and 2000. Changes in sleep duration were self-reported. The analysis was adjusted for race/ethnicity, baseline sleep duration, age, employment status, menopause, alcohol, smoking, diabetes family history, snoring frequency, sleep apnoea diagnosis, antidepressant use, shift work history, BMI and hypertension or hypercholesterolaemia.

After adjustment, and compared to the reference category (no change in sleep duration), an increase in sleep duration by 1 h/day and 2 h/day were associated with increase in weight (kg) (mean (95% CI); 0.31 (0.15, 0.47) and 0.47 (0.21, 0.72) for 1 and 2 h, respectively) (Cespedes *et al.* 2016*a*). However, these findings need to be interpreted within several limitations including that the sleep duration was self-reported, and the weight was reported at two timepoints (study start and end). Hence, variations in weight could not be captured. More importantly, the changes in weight described are relatively small considering the long follow-up and unlikely to be clinically meaningful. In addition, considering the age of the study population and the follow-up duration, sarcopenia might contribute to the changes in weight and the observed increase in weight might represent an underestimate of adiposity due to the lack of body composition data. The analysis also was not performed in specific sub groups such as people with obesity or people with short sleep duration at baseline (Cespedes *et al.* 2016*a*).

Hence, the evidence from epidemiological studies does not provide clear evidence regarding the relationship between changes in sleep duration and adiposity. As a result, interventional studies are needed to answer the questions whether sleep duration manipulation can reduce obesity or weight. These are discussed in section 4 of the review.

### Sleep duration and type 2 diabetes mellitus

#### Sleep duration as a risk factor for type 2 diabetes and gestational diabetes

Evidence from cross-sectional studies suggests a U-shaped association between sleep duration and T2DM. Short sleepers (5–6 h/day) have 2 times higher odds of being diagnosed with prediabetes and T2DM, while long sleepers are almost at a 60% higher odds of developing T2DM, compared to normal sleepers (7–8 h/day) (Chaput *et al.* 2007*b*, Engeda *et al.* 2013). The findings remained unchanged even after adjustment for confounders such as age, BMI and WC. Similarly, meta-analytic data show that long (>9 h/day) and especially short sleep (<6–7 h/day) are associated with increased risk of gestational DM (GDM) (Xu *et al.* 2018).

Meta-analyses of cohort studies published in the latest 10 years examining sleep duration and T2DM are summarised in Table 2. All the meta-analyses were in adults and included a very large number of participants. Sleep duration was self-reported in all the studies and the definitions of short and long sleep were variable. Almost all the studies were adjusted for BMI or another obesity measure. However, this may still not completely account for the effects of obesity in this correlation. Furthermore, this summary of current meta-analyses shows a major weakness of sleep research, which is the focus on single sleep disorder in isolation without accounting for the high possibility of coexistent sleep disorders, which could affect the outcomes. This is apparent in the adjustments details in Table 2, where there have been little adjustments for other sleep disorders and only a handful adjusted for napping. All the meta-analyses showed that short sleep duration increased the risk of T2DM and most (except one) showed that long sleep duration was associated with increased risk of T2DM. In addition, two meta-analyses showed conflicting results regarding the relationship between short sleep duration and risk of GDM, while one meta-analysis showed significant association between long sleep duration and risk of GDM (Cappuccio *et al.* 2010, Holliday *et al.* 2013, Shan *et al.* 2015, Anothaisintawee *et al.* 2016, Lee *et al.* 2017, Reutrakul *et al.* 2018, Xu *et al.* 2018).

**Table 2:** Ten years examining the relationship between sleep duration and type 2 diabetes mellitus.

Study	Number of studies	Number of participants	Age	Sleep recordings	Sleep duration	Comparator	Range of follow-up (years)	Diagnosis of T2DM	Outcome	95% CI	I^2^	Adjustment for covariates
Cappuccio *et al.* 2010	9	93,702	≥19	Self-reported	Short sleep (<7 h)	Normal (7 h)	4.2–32	Self-reported, blood test	Τ2DM: RR = 1.28	1.03, 1.60	58	1, 2, 3, 4, 5, 6, 7, 8, 9, 10, 11, 12, 19
Cappuccio *et al.* 2010	7	91,514	≥19	Self-reported	Long sleep (>8 h)	Normal (7 h)	4.2–32	Self-reported, blood test	T2DM: RR = 1.48	1.13, 1.96	37	
Holliday *et al.* 2013	10	447,124	N/A	N/A	Short sleep (<6 h)	Normal (7 h)	2–17	N/A	T2DM: HR = 1.33	1.20, 1.48	32	1, 2, 3, 4, 5, 6, 8, 13
Shan *et al.* 2015	9	395,584	≥32	Self-reported	Short sleep (<6 h)	Normal (7 h)	2.5–16	Medical records, self-reported, blood test	T2DM: RR = 1.06	1.01, 1.11	7.5	1, 2, 3, 4, 5, 6, 7, 8, 9, 10, 11, 12, 13, 14, 15, 16, 17
Shan *et al.* 2015	9	395,584	≥32	Self-reported	Short sleep (≤5 h)	Normal (7 h)	2.5–16	Medical records, self-reported, Blood test	T2DM: RR = 1.37	1.18, 1.59	57.1	
Shan *et al.* 2015	7	244,507	≥19	Self-reported	Long sleep (8 h)	Normal (7 h)	2.5–16	Medical records, self-reported, blood test	T2DM: RR = 1.11	0.97, 1.28	59	
Shan *et al.* 2015	7	244,507	≥19	Self-reported	Long sleep (≥9 h)	Normal (7 h)	2.5–16	Medical records, self-reported, blood test	T2DM: RR = 1.40	1.08, 1.80	75.8	
Anothaisintawee *et al.* 2016	14	583,263	≥18	Self-reported	Short sleep (≤5 h)	Normal (7–8 h)	2–32	Medical records, self-reported, blood test	T2DM: RR = 1.48	1.25, 1.76	N/A	1, 2, 3, 4, 5, 6, 7, 8, 9, 10, 12, 13, 14, 16, 18
Anothaisintawee *et al.* 2016	10	Ν/Α	≥18	Self-reported	Short sleep (<6 h)	Normal (7–8 h)	2–32	Medical records, self-reported, blood test	T2DM: RR = 1.18	1.10, 1.26	N/A	
Anothaisintawee *et al.* 2016	13	Ν/Α	≥18	Self-reported	Long sleep (≥9 h)	Normal (7–8 h)	2–32	Medical records, self-reported, blood test	T2DM: RR = 1.36	1.12, 1.5	N/A	
Itani *et al.* 2017	18	322,842	≥18	Self-reported, objectively	Short sleep (<7 h)	Normal (7–8 h)	1–30	Self-reported, blood test	T2DM: RR = 1.37	1.22, 1.53	53	1, 3, 4, 5, 6, 7, 8, 9, 10, 11, 12, 13, 14, 15, 16, 17, 18, 19, 20, 21, 22, 23
Jike *et al.* 2018	16	318,437	≥18	Self-reported, objectively	Long sleep (>7 h)	Normal (6–8 h)	1–34	Self-reported, blood test	T2DM: RR = 1.26	1.11, 1.43	63	

Adjustment for covariates: (1) age, (2) sex, (3) BMI or other measure of weight/adiposity, (4) physical activity, (5) smoking, (6) alcohol consumption, (7) comorbidities (hypertension, dyslipidaemia), (8) socioeconomic factors (income, health insurance status, employment status, education, marital status), (9) family history of diabetes, (10) depression and other mental diseases, (11) sleep-related disorders, (12) race/ethnicity, environmental factors (residence), (13) baseline health status, (14) caffeine consumption, (15) glucose levels, (16) occupational factors (shift work, working hours, occupational stress), (17) postmenopausal hormone use, (18) metabolic rate, (19) study or institution level, (20) insulin sensitivity, (21) napping, (22) total calorie intake, (23) dietary habits.

HbA1c, haemoglobin A1c; I^2^, heterogeneity; N/A, not available; RR, relative risk; T2DM, type 2 diabetes mellitus.

Regarding the relationship between short sleep duration and T2DM, the results were consistent in subgroup analyses by ethnicity (Shan *et al.* 2015) and sleep duration (Cappuccio *et al.* 2010, Itani *et al.* 2017). Men were more vulnerable to the relationship between short sleep duration and incident T2DM compared to women (Cappuccio *et al.* 2010, Itani *et al.* 2017). Similarly, consistency of findings summarizing the impact of long sleep on risk of developing T2DM was depicted in subgroup analyses by sex (Cappuccio *et al.* 2010, Jike *et al.* 2018), ethnicity (Shan *et al.* 2015) and sleep duration (Cappuccio *et al.* 2010, Jike *et al.* 2018).

The importance of sleep-related disorders (such as insomnia, sleep apnoea, sleep duration) as risk factors for T2DM was highlighted in a meta-analysis that examined risk factors for the development of T2DM (Anothaisintawee *et al.* 2016). This meta-analysis showed that after obesity and family history, sleep-related disorders had greater effect sizes in terms of T2DM risk compared to physical inactivity. In particular, the age, sex and BMI adjusted HRs for the developing T2DM in short (≤5 h) and long (≥9 h) sleep duration and physical inactivity were 1.45, 1.41 and 1.20, respectively (Anothaisintawee *et al.* 2016).

Interestingly, two meta-regressions found no dose-response relationship between short and long sleep duration and incident T2DM (Itani *et al.* 2017, Jike *et al.* 2018). This does not necessarily imply the absence of significant relationship but might possibly be attributed to the lack of published studies for the outcome of interest.

#### Sleep duration in patients with type 2 diabetes mellitus

Recent data from cross-sectional studies showed that short and long sleep durations were associated with diabetic retinopathy and albuminuria (Jee *et al.* 2017, Tan *et al.* 2018). Furthermore, in people with T2DM, several studies showed an association with short or long sleep duration and worse glycaemic control. A recent meta-analysis of cross-sectional studies examined the association of sleep duration on haemoglobin A1c (HbA1c) in people with T2DM (Lee *et al.* 2017). Results showed that people with habitual short sleep duration (<4.5–6 h per night) had significantly higher levels of HbA1c (mean difference: 0.23% 95% CI: 0.10-0.36) compared to normal sleep duration (reference group of 6–8 h per night). Si,milarly, long sleep (>8–9 h per night) was associated with higher levels of HbA1c (mean difference: 0.13%, 95% CI: 0.02–0.25), as well. However, not all the reported findings from the studies included in the quantitative synthesis were adjusted for confounders (obstructive sleep apnea, obesity or depression), and the observed differences are modest and potentially unimportant clinically, which represent a limitation of this meta-analysis.

#### The links between changes in sleep duration and type 2 diabetes prospectively

While short sleep duration has been shown consistently in multiple studies to be associated with increased risk of T2DM, studies examining the relationship between changes in sleep duration and incident T2DM did not support the findings of the above-discussed meta-analyses. This could be due to a true lack of effect or to specific reasons related to the populations studied and methods used. For example, these studies did not account for other sleep-related disorders which might also have an impact on the development of T2DM. The adjustment for confounders was variable and the increase in sleep duration examined in these studies was not necessary in people with short sleep duration at baseline. Summary of these studies is detailed below.

The Nurses Health Study (detailed above) showed that compared to no change in sleep duration, increased sleep by 2 h/day was associated with increase in the risk of T2DM after adjustment (detailed above) (HR (95%CI) (1.15 (1.01, 1.30), *P*  = 0.03) (Cespedes *et al.* 2016*a*). An increase in sleep duration by 1 h/day was not significantly associated with higher risk for T2DM (Cespedes *et al.* 2016*a*).

Similar findings were reported in the Whitehall II Study. An increase of 2 h or greater in sleep duration over a period of 5 years was associated with an increased risk of T2DM after adjustment for age, sex, ethnic group, employment grade, and change in BMI (OR = 1.50, 95%CI: 1.04–2.16) compared to a stable 7 h/ night sleep (Ferrie *et al.* 2015). Sleep duration was self-reported, and the diagnosis of T2DM was based on the criteria of WHO in 2006 and 2011 (Ferrie *et al.* 2015).

Another prospective cohort study, the Kailuan study, evaluated the effect of sleep changes over a biennial follow-up and the risk for T2DM. Almost 60,000 patients were included, with the reference category stable normal sleepers (7 h/ night). Participants who increased their sleep duration, more than 2 h during a period of 2 years, were more likely to develop DM after adjustment for age, sex, sleep duration at baseline, marital status, smoking status, physical activity, history of diabetes, BMI, hypertension, dyslipidaemia (HR = 1.24, 95% CI: 1.05–1.48). Sleep duration was evaluated with standardised questionnaires; T2DM diagnosis was based on the criteria of ADA in 2014 (Song *et al.* 2016).

Hence, the epidemiological data does not suggest that sleep prolongation can reduce the risk of T2DM. However, these studies had significant limitations and interventional studies are needed to answer the questions whether manipulating sleep duration can be used to prevent and reduce the burden of T2DM. The age of the study population in the Nurses Health Study and the Whitehall II study might have contributed to these studies findings considering the higher risk of T2DM during the follow-up due to older age and the potential development of sarcopenia and the coexistence of other sleep disorders (such as sleep apnoea) which has not been accounted for in the analysis.

## Intervention studies

In this section, we describe whether sleep manipulation is feasible and the impact of sleep manipulation on obesity and/or T2DM, with particular emphasis on sleep manipulation using non-pharmacological approaches.

### Can sleep duration be manipulated?

Several pharmacological and non-pharmacological interventions, aiming to improve sleep duration and quality, have been investigated over the years. Sleep aid drugs such as benzodiazepines, sedative antihistamines, barbiturates and melatonin analogues are widely prescribed (Chong *et al.* 2013). However, long-term use of hypnotics has been associated with serious adverse events like increased risk of death and cancer (Kripke *et al.* 2012). On the other hand, non-pharmacological interventions including the use of behavioural or psychological strategies have also been attempted. Sleep extension or restriction, sleep education, sleep hygiene education and cognitive behavioural therapy have all been studied (Koch *et al.* 2006, Mindell *et al.* 2006, Langford *et al.* 2012, Douglas & Hill 2013, Halal & Nunes 2014, Meltzer & Mindell 2014, Knowlden *et al.* 2016, Salm & Balfour 2016, France *et al.* 2018). The sleep hygiene education includes interventions such as changes in sleep environment, consistent bed- and wake-up time, avoiding behaviours affecting sleep (e.g. watching television in the bed) and activities during day helping the sleep onset (Halal & Nunes 2014).

Sleep hygiene education was found to be effective in prolonging sleep duration in people with short sleep in the study by Tasali *et al.* (2014). In short, sleepers who have overweight but otherwise healthy (ten adults, <6.5 h per night) were studied for 2 weeks and personalised sleep hygiene tips were provided from a specialist, at the first day of the intervention. At the end of the intervention, the mean sleep duration of the participants (based on actigraphy) increased from 5.6 ± 0.1 h to 7.1 ± 0.1 h (*P* < 0.01) in real life settings (Tasali *et al.* 2014). This was associated with a 4% decrease in overall appetite (*P* = 0.030) and 62% decrease in desire for sweet and salty foods (*P* = 0.017).

In a free-living, 4-weeks, parallel-design RCT study, 42 healthy adults with normal weight and short sleep duration (<7 h/night) were randomised to either maintaining their sleep duration or sleep extension by following sleep hygiene education, including the importance of sleep, current sleep recommendations (7–9 h) and the concept of sleep hygiene, from a health psychologist. Sleep was also assessed objectively by wrist actigraphy. At the end of the study, the intervention group had an increase in time-in-bed (55 min), sleep period (47 min) and sleep duration (21 min) compared to the control group (Al Khatib *et al.* 2018). This resulted in favourable changes to energy intake similar to that observed in the study by Tasali *et al.* including reduced intake of free sugars, fat, and carbohydrates.

Hence, these studies showed that sleep extension using simple non-pharmacological interventions is feasible and can be examined in RCTs.

### Sleep extension to prevent and treat obesity

Limited number of studies examined the impact of sleep manipulation on weight or adiposity. These studies largely had small sample sizes and were of short duration but they provide the basis to conduct large RCTs.

A recent systematic review provided a summary of seven studies examining the impact of sleep extension interventions on cardiometabolic risk factors (Henst *et al.* 2019). Changes in anthropometric measures, namely BMI, weight, WC and percentage body fat, were investigated in three studies. The results were consistent among studies, as no significant changes were documented in anthropometric parameters.

In two of those studies (Leproult *et al.* 2015, Al Khatib *et al.* 2018), sleep extension in people with habitual short sleep did not result in weight loss, but these studies were of short follow-up (4–6 weeks). Similarly, in a RCT including 22 people with pre- or stage 1 hypertension and short sleep duration (<7 h/day), patients were randomised to maintaining their sleep habits or a sleep extension group (sleep hygiene counselling). The overall sleep time was increased by 31 (± 9) min, but there was no effect on BMI (mean difference = 0.2, *P*  = 0.14) over 6 weeks (Haack *et al.* 2013). Additionally, no impact of sleep extension on total body fat and WC was reported (Haack *et al.* 2013, Al Khatib *et al.* 2018). The lack of effect of sleep manipulation on improving adiposity outcomes could be due to a true lack of effect, but it could be related to the study methods as the participants recruited were not overweight or obese at baseline and studies were short-term.

The aforementioned systematic review also summarised studies focusing on other outcomes that predispose to obesity, such as dietary intake and appetite. Evidence from two RCTs reporting on dietary intake is conflicting (Haack *et al.* 2013, Al Khatib *et al.* 2018). In a RCT, sleep extension in healthy participants over 6 weeks, resulted in reduction in free sugar intake (9.6 g/day, 95% CI: −16.0, −3.1), compared to the control group in which the intake remained unchanged (Al Khatib *et al.* 2018). Moreover, the percentage of daily calorie intake from protein significantly increased from baseline between the intervention and control group (mean difference 3.4 %, 95% CI: 0.6, 6.2). A trend towards lower fat and carbohydrate consumption compared to the control group was also reported in this study. On the other hand, in the RCT by Haack *et al.* (described above) sleep extension did not have an impact on dietary intake of protein, fat and carbohydrate intake (Haack *et al.* 2013). In another study without a control arm by Tasali *et al.* (described above) sleep extension in ten adults with obesity and short sleep duration was associated with a decrease in appetite by 4% and desire for sweet and salty products by 65% (measured with validated visual analog scales), while desire for fruits and vegetables remained unchanged (Tasali *et al.* 2014).

Despite the above conflicting results, studies and trials in people with obesity or overweight who were receiving a weight loss intervention suggest that sleep extension may have a role to play in terms of improving weight and fat loss. Two observational studies detailed above showed that short sleep was associated with less fat loss during calorie restricted diet and greater weight regain following bariatric surgery (Chaput *et al.* 2012, Zuraikat *et al.* 2019).

In a RCT, 49 patients with overweight or obesity (~50% in the age group of 40–64 years and BMI class of 35–40 kg/m^2^, ~85% women) were randomised to cognitive behavioral therapy (CBT) or CBT with better sleep intervention and were followed-up over 12 weeks. People who received better sleep intervention participated in a curriculum of dietary-, exercise- and sleep-related topics with the goal to embrace healthier sleep habits and hence weight reduction. The mean percentage weight loss was greater in the intervention group compared to CBT alone (5% vs 2%, *P*  = 0.04) (Logue *et al.* 2012).

In a two-conditions crossover RCT, ten patients who had overweight were put on 14 days of moderate calorie restriction (based on 90% of the resting metabolic rate) and were randomised to 8.5 h/night vs 5.5 h/night sleep duration. Sleep duration was objectively assessed (polysomnography) in the laboratory environment. There was no difference in weight loss between the two sleep durations, which is not surprising considering the short study duration and the sample size. However, the 5.5 h/night was associated with less fat mass loss and greater fat-free mass loss compared to the 8.5 h/night. Furthermore, sleep restriction was accompanied by increased ghrelin and hunger and decreased sympathetic activity and resting metabolic rate (Nedeltcheva *et al.* 2010).

Hence, there is a need for well-conducted RCTs to assess the impact of sleep manipulation on adiposity in people with obesity and short sleep duration receiving weight loss interventions as well as studies to examine the role of sleep manipulation in obesity preventions.

### Sleep extension to prevent and treat type 2 diabetes mellitus

There is very little literature regarding the impact of sleep manipulation on T2DM risk or diabetes-related outcomes in people with DM.

An uncontrolled study of 16 participants (median (IQR) age 25 (23, 27.8) years, BMI 20.8 (19.2, 23.9) kg/m^2^, ≈20% men, self-reported sleep duration <7 h/night) investigated sleep extension in real life and its impact on glucose metabolism. Sleep extension was scheduled individually, according participant’s lifestyle (habitual sleep/wake cycle, work schedules, and hobbies), but all were instructed to avoid exercise <2 h before bedtime. The study contained two phases: 2 weeks of habitual time in bed followed by 6 weeks during which participants were instructed to increase their time in bed by 1 hour per day. Sleep was assessed using self-reporting and actigraphy. Sleep time increased significantly during/by study end mainly during weekdays (self-reported: from 6.3 ± 0.5 h/night to 7.4 ± 0.7 h/night (*P* < 0.0001); actigraphy: sleep duration increased by 44 ± 34 min (*P* < 0.0001)). There was no significant difference between pre- and post-intervention in fasting glucose and insulin levels. However, the percent changes in sleep duration (based on polysomnography) correlated significantly with the percent changes in fasting glucose (*r* = 0.53, *P*  = 0.041) and insulin levels (*r* = −0.60, *P*  = 0.025). In addition, sleep extension was associated with improvement in insulin sensitivity (QUICKI *r* = 0.76, *P*  = 0.002) (Leproult *et al.* 2015).

As acute sleep restriction is capable to worsen insulin sensitivity in normal sleepers, Killick *et al.* investigated the effect of sleep recovery. Specifically, a randomised, crossover study was conducted and a total of 19 healthy males (28.6 ± 2 years old, BMI 26 ± 0·8 kg/m^2^) underwent two of the three following conditions: Friday night to Monday morning of (A) 10 h in bed each night, (B) 6 h in bed each night or (C) 10 h in bed with slow wave sleep suppression by acoustic stimuli each night. Two weeks prior to a study weekend visit, subjects were asked to keep their regular ‘catch‐up’ sleep‐wake schedules at home and records were conducted with both actigraphy and diaries. The study was performed in laboratory setting, with room lights on and offs to guide the participants to wake or sleep, while polysomnography was recorded. Meal times, quality and quantity of the food were standardised in each intervention. Insulin sensitivity was significantly increased following three nights of 10 h sleep compared to continuing sleep restriction (6 h) (8.6 vs 1.1 × 10^4^/min/μU/mL (*P*  = 0.03)). There were no significant differences between 10 h in bed with slow wave sleep suppression and either 10 h (*P*  = 0.17) or 6 h (*P*  = 0.6). However, taking into account that the study was laboratory based, these findings may not be applicable in real life conditions (Killick *et al.* 2015).

The data summarised above suggest that sleep extension might play a role in improving insulin sensitivity and hence might play a role in T2DM prevention. However, this evidence has significant limitations related to sample size, follow-up duration and generalisability of the findings. Hence, there is a need for RCTs to examine sleep manipulation as a strategy to prevent T2DM and its impact on glycaemic control and other metabolic and vascular outcomes in patients with T2DM.

## Mechanisms

**Figure 1:**
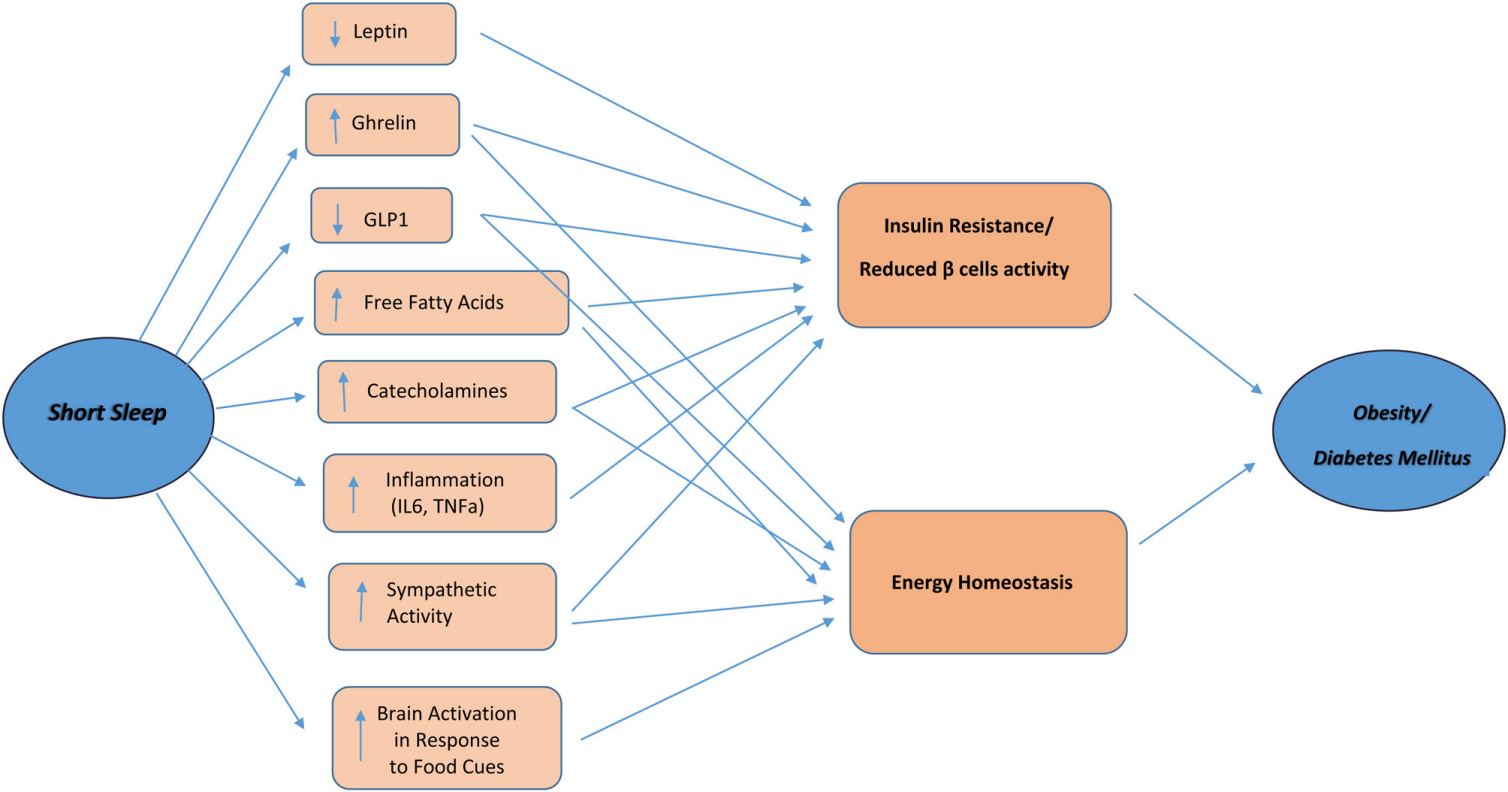
The mechanisms between short sleep duration and type 2 diabetes mellitus and obesity.

In this section, we discuss the potential mechanisms linking sleep duration to obesity and T2DM. There are multiple plausible mechanisms that lead to changes to energy balance, beta cell function and insulin resistance (Fig. 1).

### Changes to energy intake and expenditure

Population-based and experimental studies showed that short sleep duration or partial sleep deprivation are associated with increased hunger and increased appetite which are reversed with sleep extension (Spiegel *et al.* 2004, Knutson *et al.* 2007, Zhu *et al.* 2019). This was also confirmed in a recent systematic review (Zhu *et al.* 2019). This systematic review also showed that partial sleep restriction is also associated with increased calorie intake (259 kcal/day, 95%CI, 59.08–446.53). The increased appetite seems to be particularly related to higher glycaemic index, glycaemic load (particularly consumption of significantly more desserts and sweets) and carbohydrate intake although other studies also showed increased appetite for fat intake (Beebe *et al.* 2013, Yang *et al.* 2019, Zhu *et al.* 2019). Some studies also showed that short sleep duration is associated with less physical activity (Schmid *et al.* 2009). However, a recent meta-analysis showed that total and partial sleep restriction had no impact on resting or total energy expenditure including some studies using doubly labelled water method (Zhu *et al.* 2019). Also, the study of Patterson* et al.* showed that compared to those sleeping ≤6 h/day, adults who reported ≥9 h sleep consumed 178 fewer kcal/day. Compared to those with ≤6 h/day of sleep, adults who reported 9 h of usual sleep expended 113 fewer kcal/day in physical activity (Patterson *et al.* 2014).

So overall, the links between short sleep duration and obesity seem to be related to an impact on energy consumption rather than energy expenditure.

The mechanisms linking short sleep to increased energy consumption are multiple including changes in the hormones that regulate energy homeostasis (detailed in the next section). In addition, people with short sleep duration have ‘more awake hours’ available which are likely to lead to positive energy balance. Another important link between short sleep and increased energy intake is the impact of sleep restriction or short sleep on brain activity in response to food cues. Sleep restriction is associated with greater activation of brain networks involved in reward (Zhu *et al.* 2019). Sleep restriction also reduced cognitive control and activity in cortical brain regions leading to selection of foods most capable of triggering weight gain (Greer *et al.* 2013).

### Hormonal changes

Habitual short sleep duration has been shown to be associated with increased ghrelin/leptin ratio (Taheri *et al.* 2004, Chaput *et al.* 2007*a*, Knutson *et al.* 2007, Lin *et al.* 2020). Indeed, the latest meta-analysis at the field showed that participants with short sleep duration had 14% higher levels of ghrelin compared to participants with normal sleep duration (Lin *et al.* 2020). Sleep restriction found to be associated with higher levels of endocannabinoids (Jager & Witkamp 2014). The endocannabinoids system (eCB) governs food intake and hedonic hunger, leading to food overconsuming, positive energy balance and weight gain (Reutrakul & Van Cauter 2018). Furthermore, evidence indicates that eCB overactivation may lead to ß-cell dysfunction and thus, T2DM (Gruden *et al.* 2016). Experimental sleep restriction was also associated with reduction in glucagon-like peptide-1 (GLP-1) in women (St-Onge *et al.* 2012). These changes in leptin, ghrelin and GLP-1 can contribute to the increase appetite and hunger observed in people with habitual short sleep duration and/or forced sleep restriction.

The impact of sleep on cortisol secretion is another plausible mechanism linking sleep duration to obesity and T2DM. Glucocorticoids secretion is increased mainly around the onset of awaking and dropped down during sleep (Balbo *et al.* 2010). This pattern found to be different in chronic sleep restriction, with levels to be higher during evening,(Guyon *et al.* 2014), an increase in the nadir levels, a delay in the phase of the cortisol rhythm and a lower rate of decline (Rao *et al.* 2021). All these could lead to disturbance of the glucose-insulin metabolism, substrate oxidation and obesity. On the other hand, long sleep acts favourably protecting older adults from exhibiting increases in diurnal cortisol secretion over time (Rueggeberg *et al.* 2012).

Inflammation plays a role in the pathogenesis of T2DM and can affect both insulin resistance and secretion (Akash *et al.* 2013). Short sleep duration has been associated with increase inflammatory cytokines such as interleukin-6 and tumour necrosis factor a (Vgontzas *et al.* 2004, Patel *et al.* 2009, van Leeuwen *et al.* 2009). Short sleep duration was found also to be associated with elevated CRP, gamma-glutamyl transferase, uric acid and vitamin A levels but lower bilirubin, carotenoids and vitamin C, D and E levels. This observation shows once again the important role of sleep duration in inflammation and oxidate stress.

There is a relationship between sex hormones and sleep duration. Women reporting night/shift work had lower testosterone relative to women employed without night/shift work, while for every hour increase in daily sleep duration, mean estradiol concentrations increased by 3.9% and luteal phase progesterone by 9.4% (Michels *et al.* 2020). These changes could contribute to the links between menopause and obesity (Sørensen 2002).

### Insulin secretion and resistance

Experimental studies showed that sleep restriction was associated with reduced insulin sensitivity without compensatory increase in insulin secretion suggesting impaired β-cell function in people without T2DM (Morselli *et al.* 2010, Zhu *et al.* 2019). However, these associations seems to be mainly present in men rather than women, in fact in women, short sleep duration has been associated with improved insulin sensitivity (Wong *et al.* 2015, Rutters *et al.* 2016). There are no clear explanations for these gender differences, but interestingly, the links between short sleep duration and the risk for developing T2DM were much stronger in men vs women in a large meta-analysis (Itani *et al.* 2017). The reduction in insulin sensitivity has been shown to be mainly related to peripheral rather than hepatic insulin resistance (Rao *et al.* 2015). Insulin resistance may also be the result of abnormal adipocyte function, as four nights of sleep restriction resulted in approximately 30% reduction in cellular insulin signaling in adipocytes (Broussard *et al.* 2012).

The impact of sleep duration on insulin sensitivity and β-cell function can be due to multiple mechanisms. Habitual short sleep duration and experimental sleep restriction are associated with sympathetic predominance either to reduced vagal tone or increased sympathetic activity (Castro-Diehl *et al.* 2016, van Leeuwen *et al.* 2018). Increased sympathetic activity can lower response of β-cells to glucose and reduce insulin sensitivity (Bloom *et al.* 1978, Kahn *et al.* 2006, Faber *et al.* 2020).

A recent meta-analysis has examined the relationship between sleep duration and leptin. Short sleepers (vs normal sleepers) had greater leptin levels in the experimental subgroup analysis (SMD = 0.19, 95% CI (0.03, 0.35)) and greater ghrelin level in the cross‐sectional subgroup analysis (SMD = 0.14, 95% CI (0.02, 0.27)) (Lin *et al.* 2020).

Experimental sleep restriction is also associated with increased free fatty acids, which can lead to insulin resistance and hyperglycaemia by increasing gluconeogenesis (Chen *et al.* 1999, Boden *et al.* 2001, Broussard *et al.* 2015). In addition, sleep restriction is associated with several hormonal changes that can have an impact on insulin resistance and secretion including prolonged nocturnal growth hormone secretion as well as early increased morning noradrenaline and adrenaline (Broussard *et al.* 2015). Short sleep duration (experimental and habitual) has been associated with activation of the hypothalamic pituitary adrenal axis (Plat *et al.* 1999, Abell *et al.* 2016, Walsh *et al.* 2019).

## Conclusion

Short sleep duration is an independent risk factor for the development of obesity and T2DM. Short sleep also have been shown to worsen the outcomes of weight loss treatments in small studies. Changes in sleep duration had favourable impact on weight in some but not all studies and had no beneficial impact on the risk of T2DM. Several plausible mechanisms might explain the links between sleep, obesity and T2DM via changes in energy homeostasis, insulin resistance and beta-cell function.

Several studies showed that sleep manipulation is achievable. Whether sleep manipulation can prevent obesity or T2DM is currently unknown and needs to be examined. Early evidence suggests that sleep manipulation has favourable impact on weight loss or body composition as a part of lifestyle intervention (calorie restriction), but well-conducted long-term RCTs are needed to examine the role of sleep manipulation during weight loss treatments (lifestyle, pharmacotherapy, bariatric surgery). Similarly, the impact of sleep manipulation in patients with T2DM needs to be examined.

## Supplementary Material

Supplementary Materials

## Declaration of interest

Dr Tahrani reports grants from Novo Nordisk, personal fees from Novo Nordisk, non-financial support from Novo Nordisk, personal fees from Eli Lilly, non-financial support from Eli Lilly, personal fees from Janssen, personal fees from AZ, non-financial support from AZ, non-financial support from Impeto medical, non-financial support from Resmed, non-financial support from Aptiva, personal fees from BI, non-financial support from BI, personal fees from BMS, nonfinancial support from BMS, personal fees from NAPP, non-financial support from NAPP, personal fees from MSD, non-financial support from MSD, personal fees from Nestle, personal fees from Gilead, grants from Sanofi, and personal fees from Sanofi outside the submitted work. A A T is currently an employee of Novo Nordisk. This work was performed before A A T becoming a Novo Nordisk employee and Novo Nordisk had no role in this project. The other authors have nothing to disclose.

## Funding

This work did not receive any specific grant from any funding agency in the public, commercial, or not-for-profit sector.

## Author contribution statement

CA and GK drafted the manuscript. CA, GK, KN and AAT critically appraised the paper. KN and AAT proposed the idea. MS, KN and AAT gave final suggestions. All authors contributed to the final approval of the manuscript.
