# Supplementary material for: The links between sleep duration, obesity and type 2 diabetes mellitus
Source: J Endocrinol. 2021 Nov 12;252(2):125–41. doi: 10.1530/JOE-21-0155 (PMC8679843; doi:10.1530/JOE-21-0155)
Supplement: Supplementary Materials [file supplementary_material.pdf]

Supplemental Content 1. PICO question for the systematic review.

Population: any population,

Intervention: sleep duration,

Comparison: sleep duration,

Outcome: T2DM or Obesity

Supplemental Content 2. Search strategy using Medline and Cochrane Library to identify studies with sleep duration and diabetes mellitus.

MEDLINE search strategy

via pubmed

#1:

1 diabetes mellitus

2 type 2 diabetes

3 type 1 diabetes

4 exp Diabetes

5 1 or 2 or 3 or 4

6 sleep duration

7 long sleep

8 short sleep

9 sleep efficiency

10 6 or 7 or 8 or 9

11 HbA1c

12 hemoglobin A1c

13 Glycaemic control

14 Mechanism

15 Pathophysiology

16 11 or 12 or 13 or 14 or 15

(diabetes mellitus OR type 2 diabetes OR type 1 diabetes OR exp Diabetes) AND (sleep duration OR long sleep OR short sleep oral OR sleep efficiency) AND (HbA1c OR hemoglobin A1c OR glycaemic control OR mechanism OR pathophysiology)

#2: ("Diabetes Mellitus, Type 2"[Mesh]) AND "sleep duration" [Supplementary Concept]

CENTRAL search strategy

#1: MeSH descriptor: [Diabetes Mellitus, Type 2] explode all trees ()

#2: sleep duration

#3: #1 and #2

Supplemental Content 2. Search strategy using Medline and Cochrane Library to identify studies with sleep duration and obesity.

MEDLINE search strategy

via pubmed

#1:

1 obesity

2 obese

3 overweight

4 1 or 2 or 3

5 sleep duration

6 long sleep

7 short sleep

8 sleep efficiency

9 5 or 6 or 7 or 8

10 weight

11 body mass index

12 BMI

13 Mechanism

14 Pathophysiology

15 10 or 11 or 12 or 13 or 14 ()

(obesity OR obese OR overweight) AND (sleep duration OR long sleep OR short sleep oral OR sleep efficiency) AND (weight OR body mass index OR BMI OR mechanism OR pathophysiology)

#2: ("Obesity"[Mesh]) AND "sleep duration" [Supplementary Concept]

CENTRAL search strategy

#1: Obesity

#2: sleep duration

#3: #1 and #2
